# Community acquired methicillin-resistant Staphylococcus aureus pneumonia leading to rhabdomyolysis: a case report

**DOI:** 10.1186/1757-1626-3-61

**Published:** 2010-02-14

**Authors:** Poorani Nallam Goundan, Anurag Mehrotra, Deepa Mani, Indumathy Varadarajan

**Affiliations:** 1 Department of General Medicine, Sri Ramachandra University, Chennai, India

## Abstract

Community-acquired methicillin resistant Staphylococcus aureus (CA-MRSA) is considered an underreported entity in India. In this case report, the authors describe a thirty-five year old immunocompetent male presenting with severe respiratory distress requiring intubation. On further work up, a CT thorax showed features consistent with necrotizing pneumonia. The morphology and sensitivity pattern of the organism found in the bronchoalveolar lavage fluid and blood culture were consistent with MRSA. The patient's stay in the hospital was complicated by acute renal failure due to rhabdomyolysis with CPK levels of 9995 U/L. The patient was started on dialysis and improved there after. This case brings to light that CA-MRSA is becoming a problem in developing nations where antibiotics are frequently used empirically with little laboratory guidance. It also is a rare reporting of rhabdomyolysis due to CA-MRSA.

## Introduction

CA-MRSA commonly causes skin and soft tissue infections. In the current case report, we describe community acquired pneumonia due to MRSA which is until now infrequently seen. Further, this patient's clinical course was complicated by rhabdomyolysis which is even more uncommon.

## Case Report

A 35 year old South Indian male presented to the emergency department in a drowsy state with a history of high grade fever for 5 days and cough with mucoid expectoration for 3 days. The patient gave history of a thorn prick on his right hand a few days previously. He was not a know case of diabetes, hypertension or previous tuberculosis. He was a non-smoker and non alcoholic. He and his relatives denied any illicit intravenous drug use. There was no history of surgery or hospitalization in the past 10 years and no history of trauma, other than the thorn prick.

**Figure 1:**
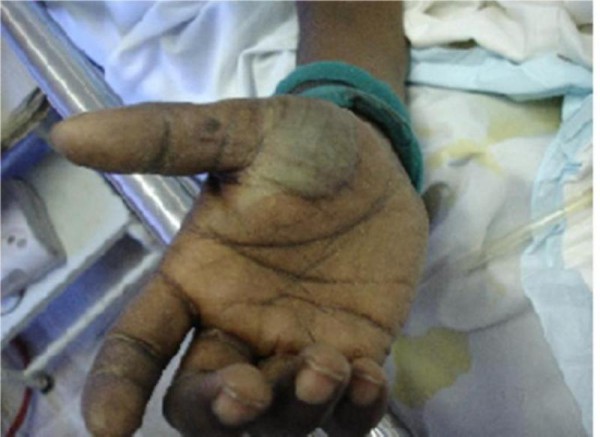
**Abscess over the thenar eminence of the right hand**.

On examination, in addition to drowsiness, the patient was found to be febrile and tachypneic. An abscess was present over the thenar eminence of the right hand which corresponded to the site of the thorn prick [Figure 1]. Systemic examination was significant for bilateral crackles over the lung fields.

**Figure 2:**
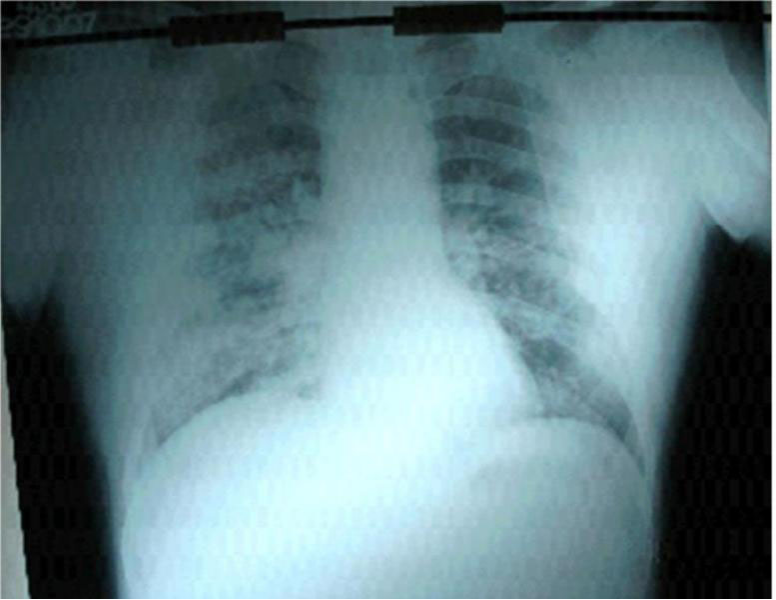
**Chest x-ray showing bilateral diffuse non-homogenous opacities**.

His Arterial Blood Gas analysis (ABG) showed hypoxemia with PO2/FIO2 < 200. Chest x-rays showed bilateral diffuse non-homogenous opacities [Figure 2], following which a diagnosis of community acquired pneumonia was made. His cardiac functions, as assessed by a surface 2D echocardiogram (2D-echo) showed normal systolic and diastolic functions. Hence, with this acute presentation, bilateral infiltrate, normal cardiac functions and PO2/FIO2 < 200, the patient was diagnosed to have acute respiratory distress syndrome (ARDS).

The patient, who was subsequently intubated in view of falling oxygen saturations was monitored in the intensive care unit and started empirically on intravenous piperacillin and tazobactum (according to the hospital policy on empirical antibiotics for severe community acquired pneumonia based on the local pattern of infecting organisms and sensitivity pattern).

His admission labs [Table 1] showed his total leukocyte counts were 2500 cells/mm3 with a polymorphic predominance, and tests for HIV as well as HbsAg were negative.

**Table 1:** Admission lab results

Parameters	Values
Hb	15 gm/dl
PCV	52.7%
TC	2500 cells/mm3
DC	P80/L19/E1
Platelets	2.43 lakh cells/mm3
ESR	3 mm/hr
BUN	13 mg/dl
Sr. Cretinine	1.4 mg/dl
Sr. Electrolytes	
Na+	139 mmol/L
K+	3.5 mmol/L
HCO3-	20 mmol/L
Cl-	95 mmol/L
Total Bilirubin	1.3 mg/dl
Direct Biliruin	0.6 mg/dl
SGPT	34 IU/L
SGOT	38 IU/L
Total Protein	7.2 g/dl
A/G ratio	3.8/3.2
Urine Analysis	Normal

**Figure 3:**
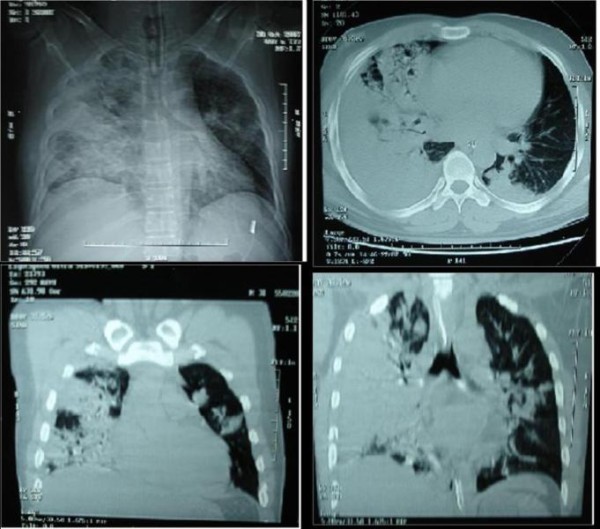
**CT thorax showing features suggestive of necrotizing pneumonia**.

Two surface 2D echos and one transesophageal echo showed no evidence of infective endocarditis. Following up with the diagnosis of bilateral bronchopneunomia with ARDS, a CT thorax was ordered for the patient, which revealed necrotizing pneumonia [Figure 3]. Serial Chest X-Rays were taken there after to monitor the patient.

The bronchoalveolar lavage (done prior to intubation) and two blood cultures (from two different sites drawn at two different times) were positive for MRSA (sensitive only to vancomycin and linezolide). The patient's antibiotics were changed to vancomycin and meropenam (as per glomerular filtration rate calculation using Cockroft and Gault formula). Meropenem was added as an empirical gram negative cover.

On day 12 of hospitalization, the patient developed a decreased urine output, with a BUN (blood urea nitrogen) of 114 mg/dl, serum creatinine of 5.8 mg/dl and CPK (creatine phosphokinase) of 9995 U/L. The patient's urine was positive for myoglobin and a diagnosis of rhabdomyolysis was made. Hemodialysis was initiated. Vancomycin was changed to Linezolide 600 mg twice a day and continued for 6 weeks till two cultures were negative.

Following this, the patient improved and was discharged with a serum creatinine of 3.7 and with advice regarding continuing hemodialysis on an outpatient basis, respiratory rehabilitation and physiotherapy and tracheostomy care.

## Discussion of the Case

Methicillin resistant Staphylococcus aureus (MRSA) is growing in prevalence in the Indian scenario. Studies form different centers around the country estimate the prevalence to be between 20 and 40% and sometimes even higher [1,2].

While MRSA has traditionally been acquired nosocomially, the occurrence of community acquired MRSA infection has recently begin to pose a threat to health care. By definition, hospital acquired infections are diagnosed when they develop after 48 hours of hospitalization and community acquired infections when they develop before that period.

One study in India, suggests that the incidence of CA-MRSA is low enough to consider it unwarranted to treat community acquired infections with antibiotics that cover MRSA [3].

CA MRSA have been found to cause skin and soft tissue infections more commonly then when compared to HA (hospital acquired)-MRSA [4]. They are also associated with certain exotoxin genes (like the Panton Velentine leukocidin gene) not usually seen in nosocoimally acquired stains [5]. In a study comparing invasive CA-MRSA with those causing skin and soft tissue infections, the latter was more commonly seen in male patients with a history of underlying conditions (immunosuppressive therapy, emphysema/COPD, injection drug use and smoking) [6].

Rhabdomyolysis is described as the dissolution and disintegration of striated muscle, that can result in an acute, potentially fatal clinical syndrome. Acute renal failure occurs as a result of renal vasoconstriction, heme-protein induced toxicity and intraluminal cast formation. However, with out the presence of aciduria and hypovolemia along with the heme-protein, it would not have its nephrotoxic effect [7].

Infections form a part of the long list of etiologies of rhabdomyolysis. Respiratory infection appears to the chief contributor among the infectious causes of rhabdomyolysis [8] Rhabdomyolysis in CA-pneumonia is more commonly associated with Legionella, Influenza virus, Streptococcus pneumoniae, Chlamydia psttaci and Mycoplasma [9]. Our patient had a thorn prick in his hand which turned into an abscess. This could have been the source for Staphylococcus aureus infection. He was not hospitalized or hadn't visited the hospital in the previous several years. In addition, the two blood cultures drawn at two different sites under sterile precautions at the time of admission were positive for MRSA and the bronchoalveolar lavage taken just prior to intubation showed MRSA. Hence his infection was community acquired and not hospital acquired.

In a study of 41 patients with community-acquired pneumonia admitted in the ICU, the 29% who had had an elevated CPK (more than 1000 U/l) had higher mortality when compared to the 71% without elevated CPK, though the initial severity and renal impairment was the same for the two groups. This suggests that the presence of rhabdomyolysis is a bad prognostic factor irrespective of the presence or absence of renal impairment [10].

The diagnosis of rhabdomyolysis mandates measures to prevent renal impairment. The patient should be adequately hydrated and monitored for urine output, electrolyte balance and acid-base status. The onset of oligouric renal failure, persistent electrolyte and pH disturbance and other related complications requires the initiation of dialysis.

## Conclusion

Patients with severe community acquired pneumonia should not be excluded from suffering from CA-MRSA due to the growing problem of drug resistant bacteria beyond the confines of the hospital. In addition, respiratory infections are a common cause of rhabdomyolysis, which should be considered with a high index of suspicion in order to initiate timely intervention.

## List of abbreviations

CA-MRSA: community acquired - methicillin resistant Staphylococcus aureus; ARDS: acute respiratory distress syndrome; ABG: arterial blood gas (analysis); Echo: echocardiogram; HIV: human immunodeficiency virus; CT: computerized tomography; BUN: blood urea nitrogen; CPK: creatine phosphokinase.

## Consent

Written informed consent was obtained from the patient for the publication of this case report and accompanying images. A copy of the written consent is available for review by the Editor-in-Chief of this journal.

## Competing interests

The authors declare that they have no competing interests.

## Authors' contributions

PNG, AM, DM and IV played various parts in the patient care, acquisition of data, analysis and interpretation of data, review of literature, drafting and revising the manuscript. All authors have read and approved the final manuscript.
